# Impact of Multiple Mechanical Recycling Cycles via Semi-Industrial Twin-Screw Extrusion on the Properties of Polybutylene Succinate (PBS)

**DOI:** 10.3390/polym17141918

**Published:** 2025-07-11

**Authors:** Vito Gigante, Laura Aliotta, Luigi Botta, Irene Bavasso, Alessandro Guzzini, Serena Gabrielli, Fabrizio Sarasini, Jacopo Tirillò, Andrea Lazzeri

**Affiliations:** 1 Department of Civil and Industrial Engineering, University of Pisa, 56122 Pisa, Italy; vito.gigante@unipi.it (V.G.); andrea.lazzeri@unipi.it (A.L.); 2 Department of Engineering, University of Palermo, 90128 Palermo, Italy; luigi.botta@unipa.it; 3 Department of Chemical Engineering Materials Environment, Sapienza University of Rome, 00184 Roma, Italy; irene.bavasso@uniroma1.it (I.B.); fabrizio.sarasini@uniroma1.it (F.S.); jacopo.tirillo@uniroma1.it (J.T.); 4 ChIP Building, School of Science and Technology, University of Camerino, 62032 Camerino, Italy; alessandro.guzzini@unicam.it (A.G.); serena.gabrielli@unicam.it (S.G.)

**Keywords:** polybutylene succinate (PBS), mechanical recycling, production scraps, extrusion, injection molding

## Abstract

This study investigates the effects of repeated mechanical recycling on the structural, thermal, mechanical, and aesthetic properties of poly(butylene succinate) (PBS), a commercially available bio-based and biodegradable aliphatic polyester. PBS production scraps were subjected to five consecutive recycling cycles through semi-industrial extrusion compounding followed by injection molding to simulate realistic mechanical reprocessing conditions. Melt mass-flow rate (MFR) analysis revealed a progressive increase in melt fluidity. Initially, the trend of viscosity followed the melt flow rate; however, increasing the reprocessing number (up to 5) resulted in a partial recovery of viscosity, which was caused by chain branching mechanisms. The phenomenon was also confirmed by data of molecular weight evaluation. Differential scanning calorimetry (DSC) and thermogravimetric analysis (TGA) confirmed the thermal stability of the polymer, with minimal shifts in glass transition, crystallization, and degradation temperatures during the reprocessing cycles. Tensile tests revealed a slight reduction in strength and stiffness, but an increase in elongation at break, indicating improved ductility. Impact resistance declined moderately from 8.7 to 7.3 kJ/m^2^ upon reprocessing; however, it exhibited a pronounced reduction to 1.8 kJ/m^2^ at −50 °C, reflecting brittle behavior under sub-ambient conditions. Despite these variations, PBS maintained excellent color stability (ΔE < 1), ensuring aesthetic consistency while retaining good mechanical and thermal properties.

## 1. Introduction

Bio-based and/or biodegradable polymers have increasingly gained attention as environmentally friendly alternatives to conventional plastics due to their ability to decompose under natural conditions [1]. However, biodegradability alone is insufficient to comprehensively mitigate the global plastic waste crisis; it is also important to ensure that bio-based and biodegradable polymers are also recyclable [2]. While biodegradation offers a route for natural breakdown, recycling (and above all, mechanical recycling) is a more efficient and environmentally sustainable strategy [3]. Biodegradation is often slow and dependent on variable environmental conditions, whereas recycling provides a more controlled and economically viable pathway to waste management [4]. Integrating recyclability into biodegradable polymers contributes to both environmental protection and the advancement of circular economy models [5]. Although biodegradation might be effective in specific environments such as soil or marine ecosystems, recycling offers broader, more predictable control over materials, minimizing their dispersion and preserving resources [6]. Furthermore, recycling enables the maintenance of material quality during the reuse process, thereby sustaining higher performance levels and reducing the need to extract new raw materials [7]. The need for recyclable, biodegradable polymers has become even more critical in the context of widening industrial applications. While the recycling of non-biodegradable bioplastics such as bio-polyethylene terephthalate (bioPET), bio-polyethylene (bioPE), and biopolypropylene (bioPP) is well-established, the recyclability of biodegradable bioplastics remains less explored [8]. Nevertheless, recent research indicates that many biodegradable, bio-based polymers can be successfully recycled, mechanically or chemically, with promising results [9,10]. When further reuse or recycling is no longer feasible, these materials may also be composted, contributing to soil enrichment and providing a fully circular end-of-life scenario [11]. The biodegradation of commercial bioplastics should take place in dedicated composting facilities, but this process should be considered only as the final stage of their life cycle, preceded by multiple phases of reuse and recycling [12]. While biodegradation is acknowledged as a type of recycling, commonly known as “organic recycling,” its primary focus does not lie in reclaiming plastic materials or monomers to reintegrate them into the life cycle of plastic products. In contrast, mechanical (primary or secondary) and chemical (tertiary) recycling methods are expressly crafted to fulfil this objective [13]. When biodegradable plastics are of superior quality, they can undergo primary recycling, where the recycled plastic mimics the functionality of virgin plastic, or secondary recycling, repurposing it for less demanding applications [14]. In cases where material quality falls below a specified threshold, chemical recycling becomes a viable option for recovering valuable monomers crucial for the formulation of new polymers or chemicals.

To provide a broader perspective on the sustainability of PBS end-of-life strategies, it is worth comparing the environmental impacts of mechanical recycling with those of biodegradation and industrial composting. While the mechanical recycling of PBS involves energy input during reprocessing (mainly extrusion), it generally leads to lower overall CO_2_ emissions when compared to complete biodegradation or composting, which result in the full mineralization of the polymer to CO_2_ and H_2_O, hence releasing the carbon content of the material.

Indeed, recycling bio-based plastics reduces life-cycle emissions by 25–50% versus composting, as it avoids decomposition emissions and retains material value [15,16]. Biodegradation and composting release significant CO_2_ during microbial breakdown and require energy-intensive industrial conditions [17].

For instance, Soroudi et al. [18] stress that mechanical recycling preserves the embodied energy of the polymer, while composting releases it; moreover, Rujnić-Sokele et al. [19] indicate that the energy demand for composting is lower in terms of process input but results in a complete loss of material value, whereas recycling allows multiple reuses, delaying the need for virgin polymer production.

In this context, there has been significant interest in succinate-derived polyesters such as poly(ethylene succinate) (PES), poly(propylene succinate) (PPS), poly(butylene adipate) (PBA), and poly(butylene succinate) (PBS) and its well-known copolymer, poly(butylene succinate-co-adipate) (PBSA) [20].

This paper focuses on poly(butylene succinate) (PBS) and aims to evaluate whether recycled PBS, obtained through a semi-industrial mechanical recycling process simulating production scrap recovery, can be effectively reused across multiple applications.

PBS is typically synthesized via a polycondensation reaction between a diacid (or acid anhydride) and diols, with water as a byproduct. Initially produced from petrochemical sources by Showa Highpolymer (Shanghai, China), a major advancement in PBS development has been the shift toward renewable feedstocks such as sugarcane, cassava, and corn. This transition has positioned PBS as a promising, sustainable, bio-based, and biodegradable alternative to conventional plastics [21]. From the point of view of physical properties, PBS is a white crystalline thermoplastic polymer with a density of 1.25 g/cm^3^, a melting point in the range of 90–120 °C, and a low glass transition temperature (T_g_) of about −40 °C [22].

The are several challenges in scaling the mechanical recycling of PBS. First, industrial-scale recycling requires careful control of processing parameters (temperature, shear, residence time) to minimize degradation and maintain product quality [23]. Moreover, contamination with other polymers (e.g., PET, PLA) can negatively impact the recycling stream, leading to the deterioration in the mechanical properties of recycled PBS. In fact, it has been observed in literature [24] how a small amount of PBS in PET reduces the tensile strength and modulus due to the poor miscibility between the two polymers. Consequently, effective sorting and purification steps are necessary. Another important aspect that affects PBS recycling is correlated to the variability in feedstock composition (e.g., presence of plasticizers, fillers, or blends) that can affect melt flow, crystallinity, and the final properties of recycled products, requiring robust quality control and possibly blending strategies to homogenize input streams [25]. Finally, the color of recycled PBS is influenced by the original coloration of the feedstock, degradation during use, and the recycling process itself. Unlike virgin resins, recycled PBS often exhibits color shifts, reduced transparency, or the development of off-hues due to pigment residues and thermal oxidation. Consequently, color measurement using spectrophotometry is recommended for quality assurance, as visual inspection can be subjective. Color variability may limit recycled PBS applications where aesthetics is critical unless further purification or re-coloring steps are implemented. In the last decade, the recyclability of PBS was first investigated by Georgousopoulou et al. [26], and it was found that, when PBS was reprocessed at temperatures higher than 190 °C, it resulted in branching/recombination and chain scission reactions with extrudates of higher viscosity, showing a bimodal distribution of molar masses. When stabilizers are added, the thermo-mechanical degradation of PBS can be significantly suppressed. This effect reveals the radical character of the degradation reactions. More recently, a grape pomace extract and a grape seed extract were melt-mixed with PBS [27]. The grape seed extract exhibited the best results because it maintained an unaltered PBS molecular weight both after several reprocessing steps and after 300 h of oven aging. These improvements were attributed to the presence of polyphenols and the excellent radical scavenging activity of this additive. The coating applied by the photografting of monomers was found to be a good strategy for controlling the degradability and reprocessability of PBS [28]. In this context, Kanemura et al. [29] immersed PBS in water and noticed that the bending strength of PBS decreased as the immersion time and the immersion temperature increased. This effect was attributed to the hydrolysis of PBS. The degraded PBS was then reprocessed, and an unexpected increase was observed in both the bending strength and the molecular weight of PBS. The increase was justified considering the autocatalytic action of the esterification of PBS molecules during reprocessing. Interestingly, the characteristic increase in molecular weight observed for PBS after reprocessing was in contrast to the decrease observed in poly(lactic acid) (PLA). Jbilou et al. [30] instead investigated a “green” recycling route for polybutylene succinate (PBS) based on reactive extrusion in the presence of an enzyme catalyzing the hydrolysis of this aliphatic polyester: lipase B from *Candida antarctica*. This enzyme was chosen due to its thermal stability, as reactive extrusion was performed at 120 °C. Zhang et al. [31] found that the repeated recycling of PBS led to significant degradation, including a 67% decrease in molecular weight and an increase in polydispersity. Lab-scale experiments using a twin-screw extruder at varying temperatures (120 °C and 140 °C), screw speeds (30 rpm and 100 rpm), and durations (3 to 24 h) showed that higher screw speed (100 rpm) and extended processing time (over 12 h) significantly reduced the viscosity of regenerated PBS. Finally, Nomadolo et al. [32] found that the repeated processing of PBS under aggressive conditions of shear stress and high temperatures caused chain scission, leading to increased melt flow rates and reduced molecular weight. This enhanced polymer chain mobility and crystallinity resulted in stiffer materials with higher storage modulus but reduced impact resistance and strain at breaks.

The main novelty of this study is that poly(butylene succinate) (PBS) underwent up to five mechanical reprocessing cycles using a semi-industrial twin-screw extrusion setup to assess its recyclability under conditions that better reflect potential industrial applications. In line with the literature, the practical number of mechanical recycling cycles for PBS is typically between three and five, beyond which mechanical properties degrade significantly, limiting further applications. The five cycles analyzed in this study therefore represent the upper recommended limit for PBS mechanical recycling [23]. The transition from the lab-scale to the semi-industrial extrusion of PBS introduces critical differences in process conditions that affect polymer integrity. Industrial extruders often operate under vacuum or inert atmosphere, effectively minimizing oxygen exposure and thus reducing thermo-oxidative degradation, as observed in studies on PBS melt processing [33]. Additionally, the screw design in semi-industrial setups allows for tailored shear stress distribution and residence time control through modular elements such as kneading blocks, which are not present in most lab-scale systems [34].

Following extrusion, samples were shaped via injection molding and systematically analyzed to evaluate the evolution of rheological, mechanical, aesthetic, chemical, and molecular weight properties. Particular attention was given to the behavior of highly reprocessed PBS under varying impact test temperatures, aiming to identify possible application limits and temperature-dependent performance thresholds. While prior studies have largely focused on the lab-scale degradation or chemical recycling of PBS, this work offers a broader perspective by simulating real-world mechanical recycling scenarios without the use of additives and chain extenders. The goal is to define not only the material’s recyclability, but also its functional resilience and suitability for extended use in circular economy frameworks.

## 2. Materials and Methods

### 2.1. Materials

The material used in this study is polybutylene succinate (PBS)—specifically, the bio-based trade name BIOPBS FZ71PM, sourced from Mitsubishi (Tokyo, Japan). This semi-crystalline polyester is suitable for injection molding applications. To simulate the production of recycled pellets, the PBS was subjected to five extrusion cycles using a semi-industrial twin-screw extruder (COMAC, Cerro Maggiore, Italy) equipped with two 25 mm co-rotating screws (L/D = 44), an integrated motor, and a temperature control system that utilized distilled water as a coolant.

During each extrusion cycle, the pellets were fed into the extruder via a gravimetric feeder. Upon exiting the die, the extrudate was immediately cooled in a water bath, then air-dried and cut into pellets using a pelletizer. This process was repeated for each extrusion cycle.

Following extrusion, a portion of the pellets was dried in a ventilated oven (Binder 730, BINDER, Tuttlingen, Germany) at 60 °C. The remaining pellets underwent further extrusion cycles before being stored in the same oven and subsequently tested. For analysis, pellets from the 1st, 3rd, and 5th extrusions were selected and compared. The extrusion parameters are described in Table 1 and Table 2.

From Table 2, it can be observed that, despite maintaining the same temperature profile, throughput, and screw speed, both the head pressure and motor power absorption exhibited an almost linear decrease throughout the extrusion cycles. However, a slight trend reversal was noted in the fifth extrusion. This behavior will be further discussed and contextualized in the discussion section, where it will be correlated with rheological data and molecular weight measurements.

After 1, 3 and 5 extrusion cycles, the extruded pellets were sent to a Megatech H10/18 injection molding machine (TECNICA DUEBI s.r.l., Fabriano, Italy) for the injection molding of dog-bone specimens (ISO 527–1A [35], width: 10 mm, thickness: 4 mm, useful length: 80 mm) that are useful for mechanical characterizations.

**Figure 1:**
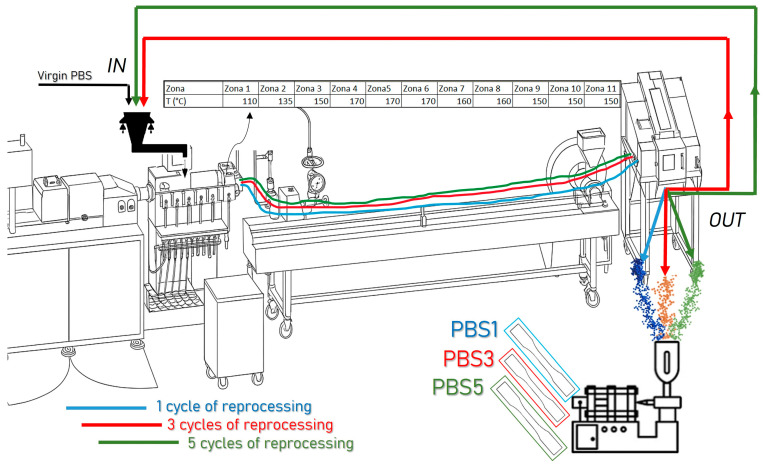
PBS reprocessing pathway.

In Figure 1, the complete pathway of the primary and secondary processing carried out in this work is summarized.

### 2.2. Characterization Methodologies

For all the methodologies applied, the extruded and molded materials were named as PBS1 (1 cycle of extrusion), PBS3 (3 cycles of extrusion) and PBS5 (5 cycles of extrusion).

#### 2.2.1. Melt Mass-Flow Rate (MFR)

The melt flow behavior of the extruded granules was evaluated using a Melt Flow Tester M20 (CEAST, Torino, Italy). The MFR was measured according to ISO 1133 standards [36] using a load of 2.16 kg at 190 °C.

#### 2.2.2. Rheological Characterization

The rheological properties of extruded and reprocessed PBS granules were investigated using a rotational rheometer (ARES G2, TA Instruments, New Castle, DE, USA) equipped with parallel plate geometry. Measurements were carried out at 170 °C under an air atmosphere using 25 mm diameter plates and a fixed gap of 1 mm. Dynamic frequency sweep tests were conducted in the angular frequency range of 0.1 to 100 rad/s to evaluate the viscoelastic behavior of the samples. The strain amplitude was set at 5%, a value confirmed to fall within the linear viscoelastic region through preliminary strain sweep experiments. All measurements were performed in triplicate.

#### 2.2.3. Molecular Weight Evaluation

The measurements of molecular weights were performed by an Agilent 1260 Infinity II Multi-Detector Suite (MDS) device (Agilent Technologies, Santa Clara, CA, USA), constituted by three different detectors (G7800A): RI vs. a dual light-scattering detector (15° and 90°), a 4-channel vacuum degasser (G7111B), an autosampler (G7129A), and a thermostatic column compartment (G7116A). The system was equipped with a guard column (Agilent GPC/SEC Guard Column) followed by two columns in series (PLgel MIXED-C and PLgel MIXED-D). The measurements were processed using Agilent GPC/SEC Software, Version A02.01. The mobile phase used was CHCl_3_, and the flow rate was fixed at 1.0 mL/min. The polystyrene standards (Mp values in the range of 580–283,800 g/mol) were used for column calibration.

#### 2.2.4. Thermogravimetric Analysis (TGA)

TGA analysis was performed using an STA 2500 Regulus instrument (Netzsch, Selb, Germany). The measurements were conducted under a nitrogen gas flow with a temperature ramp rate of 10 °C/min, ranging from room temperature to 600 °C.

#### 2.2.5. DSC Characterization

The thermal properties of PBS1, PBS3, and PBS5 were investigated by differential scanning calorimetric (DSC) analysis using a TA-Q200 DSC (TA Instruments-Waters LLC, New Castle, DE, USA). Nitrogen, set at 50 mL/min, was used as purge gas, while indium was used as a standard for temperature and enthalpy calibration. The materials used for DSC analysis were cut from the ISO 527 1-A specimens. Consequently, the thermal properties were evaluated considering only the first DSC heating run to consider the effect of the injection molding process. The following thermal program was adopted: heating ramped from −50 °C up to 200 °C at 10 °C/min, followed by an isothermal step for 1 min. The PBS melting enthalpy was determined from the corresponding peak area in the thermogram; the crystallinity percentage (*X_c_*) was calculated with Equation (1) [37]:

(1) $$X_{c}\;\left(\%\right)=\frac{\Delta H_{m}}{\Delta H_{m}^{{}^{\circ}}}\cdot 100$$

where Δ*H_m_* is the melting enthalpy of PBS detected with DSC and $\Delta H_{m}^{{}^{\circ}}$ is the theoretical melting enthalpy of 100% crystalline PBS that was taken equal to 110.3 J/g [38].

#### 2.2.6. Tensile Tests

Quasi-static tensile tests were conducted three days after injection molding, keeping the specimens inside a dry keeper (SANPLATEC Corp., Osaka, Japan) at a controlled atmosphere (room temperature and 50% of humidity). An MTS Criterion model 43 universal testing machine (MTS Systems Corporation, Eden Prairie, MN, USA) was used to perform tensile tests at room temperature. The tests were carried out with a crosshead speed of 10 mm/min and a load cell of 10 kN via an extensometer model 634.25F-54 (MTS Systems Corporation, Eden Prairie, MN, USA) with a gauge length of 50 mm, interfaced with the MTS Elite Software(MTS Testsuite version 4.1). At least five specimens were tested for each composition, and the average values were reported.

#### 2.2.7. Heat Deflection Temperature (HDT) Tests

HDT tests (which assess the temperature at which a specimen measuring 80 mm × 10 mm × 4 mm, subjected to three-point bending, deflects by 0.34 mm under a stress of 0.45 MPa) were also performed on an HVT302B (MP Strumenti, Bussero, Italy) in accordance with ISO 75–1 (method B) [39].

#### 2.2.8. Impact Tests and Further Characterizations on PBS5

The impact strength of each sample (with size 80 mm × 10 mm × 4 mm) was evaluated by Charpy impact tests (ISO 179-2) [40] in an edgewise mode (type A notch and a span of 62 mm). Tests were conducted with a CEAST/Instron 9340 instrumented drop weight tower using an impact velocity of 2.90 m/s. PBS5 samples, obtained through the maximum amount of mechanical recycling simulations of production scraps cycles, were subjected to Charpy impact tests at variable temperatures (−50 °C, 23 °C, and 50 °C). All tests were repeated at least five times and reported as mean values. Prior to testing, samples were conditioned at the test temperature for one hour.

#### 2.2.9. Color Variation

The color variation among the reprocessed molded specimens was evaluated using a Fru WR10QC digital colorimeter (Semiki Instrumentation Co., Ltd., Hanoi, Vietnam) calibrated with a standard white and black tile. At least five measurements were carried out, and the average value was then calculated. The parameter used to quantify color variation was the total color difference, (Δ*E*), which represents the Euclidean distance between two points in the CIELAB color space. In practical terms, ΔE indicates the extent of perceivable color change to the human eye: values below 1 are generally considered imperceptible under standard lighting conditions. The CIELAB color coordinates (L*, a*, and b*) were evaluated with the above-mentioned colorimeter, and the Δ*E* was shown according to Equation (2):

(2) $$\Delta E=\sqrt{\Delta L^{2}+\Delta a^{2}+\Delta b^{2}}$$

## 3. Results

### 3.1. Melt Mass-Flow Rate Results

**Figure 2:**
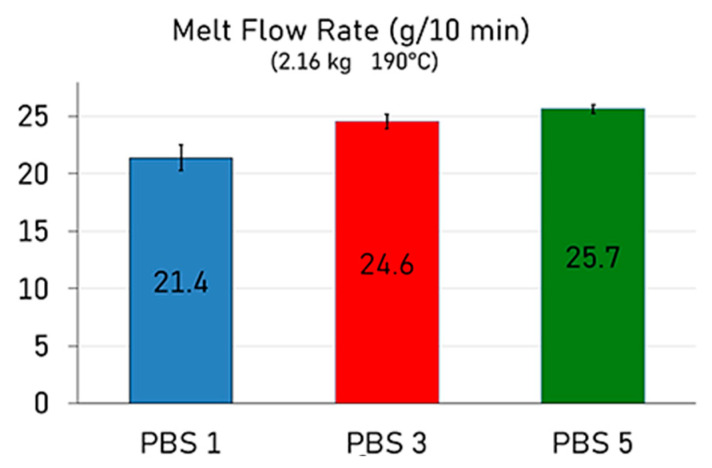
Melt mass-flow rate of reprocessed PBS.

As illustrated in Figure 2, the melt mass-flow rate (MFR) values showed an upward trend with increasing reprocessing cycles with a major increase from PBS1 to PBS3 and then a slight increment from PBS3 to PBS5. Specifically, the MFR increased from 21.4 g/10 min after the first extrusion cycle to 24.6 g/10 min following the third cycle and further to 25.7 g/10 min after five extrusion cycles. This behavior is commonly associated with the thermal and mechanical degradation of the polymer chains, which occurs due to the repeated exposure to high temperatures and shear stresses during melt processing [41]. This phenomenon is well-documented in polymer reprocessing studies and is particularly relevant for biodegradable polyesters like PBS, which are susceptible to hydrolysis and thermal degradation [42].

Nevertheless, it is worth noting that the increase in MFR between the third and fifth cycles was relatively modest, suggesting that the most significant molecular breakdown occurs within the initial processing cycles. Beyond this point, the polymer is less prone to degradation under the same processing conditions. Despite the observed increase in fluidity, the MFR values obtained after five cycles remained within an acceptable range for common secondary processing techniques, such as injection molding [43]. This indicates that PBS retains sufficient processability even after multiple reprocessing cycles, supporting its potential for use in circular manufacturing models.

### 3.2. Rheological Results

**Figure 3:**
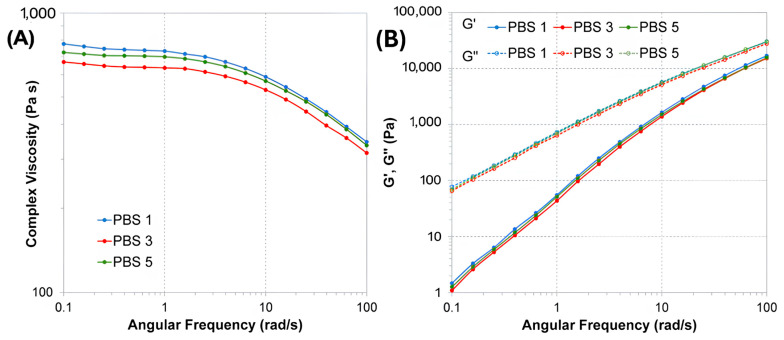
Rheological behavior of PBS samples subjected to different numbers of reprocessing cycles (PBS1, PBS3, PBS5): (**A**) Complex viscosity as a function of angular frequency; (**B**) Storage modulus (G′) and loss modulus (G″) as functions of angular frequency.

To evaluate the effects of repeated reprocessing on the rheological behavior of PBS, frequency sweep tests were performed, and the results are shown in Figure 3. In particular, Figure 3A reports the evolution of the complex viscosity (η*), while Figure 3B shows the storage (G′) and loss (G″) moduli as a function of angular frequency over the investigated range.

As expected, PBS3 exhibited a lower viscosity compared to PBS1, indicating a reduction in molecular weight due to thermomechanical degradation occurring during reprocessing. This behavior is commonly observed in aliphatic polyesters, such as PBS and PLA, which are susceptible to chain scission under repeated thermal and shear stresses [26,44,45].

Surprisingly, the sample reprocessed five times (PBS5) showed an increase in viscosity compared to PBS3 despite remaining slightly below the value of PBS1. This unexpected rise in viscosity may be attributed to chain rearrangements occurring during prolonged reprocessing, possibly involving the formation of long-chain branched structures, as reported for similar systems [46,47]. Such branching phenomena can result from intermolecular transesterification reactions, which are promoted by extended thermal exposure and may lead to the recombination of shorter chain fragments [42]. As a result, the melted PBS5 became more viscous than PBS3, although it did not fully recover the viscosity of the pristine material. It is worth noting that the observed trend in complex viscosity did not fully align with the MFR results, which showed a progressive increase from PBS1 to PBS5. This discrepancy can be attributed to the different flow regimes probed by the two techniques. While long-chain branching formed during extended reprocessing may enhance melt elasticity and increase viscosity at low shear [48], it can simultaneously promote better flow alignment under high shear stress conditions, leading to a further increase in MFR values.

These findings suggest that, while initial reprocessing cycles primarily induce degradation via chain scission, further processing may trigger secondary reactions that alter the molecular architecture, balancing or partially compensating the viscosity loss observed at intermediate cycles.

A similar trend was observed for the storage and loss moduli (Figure 3B), further confirming the effects of reprocessing on the viscoelastic properties of PBS. In particular, PBS3 exhibited the lowest values of both G′ and G″, indicating a reduction in both the elastic and viscous components of the melt, consistent with the molecular weight decrease due to chain scission. PBS5 showed a slight increase in both moduli compared to PBS3, although it remained below the levels observed for PBS1, mirroring the trend found in the viscosity data. This suggests that the partial recovery of melt elasticity and viscosity in PBS5 could be associated with structural rearrangements such as chain branching.

### 3.3. Molecular Weight Results

The results are summarized in Table 3.

The initial extrusion cycle of PBS resulted in a slight increase in number-average molecular weight (M_n_) and a decrease in weight-average molecular weight (M_w_), indicating predominant chain scission due to thermal and hydrolytic degradation. This observation aligns with the known susceptibility of aliphatic polyesters, such as PBS, to hydrolysis and thermal degradation during melt processing, particularly in the presence of residual moisture and elevated temperatures [49,50,51].

However, subsequent extrusions exhibited a notable increase in both M_n_ and M_w_. Repeated melt processing appears to induce recombination and partial cross-linking, even without the use of chain extenders. These effects likely stem from transesterification or radical-induced crosslinking, processes known to occur under prolonged thermal and mechanical stress [26,52]. The observed increase in number-average molecular weight, coupled with a decrease in the polydispersity index (PDI) after multiple extrusion cycles, lends further credence to this hypothesis. While chain scission typically broadens the molecular weight distribution, recombination and branching tend to narrow it.

It is important to highlight that the trend of the molecular weight was consistent with the rheological data, which showed an initial decrease in viscosity followed by a subsequent increase. In particular, the weight-average molecular weight exhibited this same pattern—first decreasing and then increasing—which reflected an initial chain scission process followed by branching in the PBS.

A complementary evaluation of the melt mass-flow rate revealed increasing values across successive extrusion cycles, from 21.4 g/10 min after the first extrusion to 25.7 g/10 min after the fifth cycle. This progressive increase suggests an overall enhancement in melt fluidity, typically associated with a reduction in average molecular weight due to chain scission. However, the trend may also reflect the formation of limited chain branching, which can reduce entanglement density and crystallinity, thereby increasing chain mobility and promoting melt flow. It is essential to note that moderate branching does not yet result in extensive crosslinking, which would otherwise restrict molecular motion and decrease the MFR. The observed MFR values therefore support the combined presence of chain degradation and low-level branching phenomena during reprocessing [42]. Together, the GPC and MFR results suggest an initial degradation during the first processing cycle, followed by structural rearrangements that could involve limited branching or recombination in subsequent cycles, thereby partially restoring or increasing the molecular weight while reducing the PDI.

Indeed, in the semi-industrial re-extrusion trials presented in this paper, the molecular weight and viscosity of PBS decreased over the first three cycles before partially recovering by the fifth cycle. This contrasts with continuous, long-duration lab-scale experiments [31,32], where both properties declined steadily. Differences in extruder scale, geometry, residence time, and thermal profile can account for these outcomes. A larger semi-industrial extruder features wider flow channels and a lower surface-to-volume ratio, resulting in less intense local shear [53] and less uniform heating than a lab-scale device. Lab-scale extruders expose polyesters to sustained, homogeneous shear and heat, promoting uninterrupted chain scission and oxidative degradation [54]. In contrast, semi-industrial trials involved shorter, repeated cycles that introduced thermal “pulses”; after several cycles, chain recombination or entanglement processes became more significant [47], explaining the rebound in molecular weight and viscosity by the fifth cycle.

Moreover, the thermal profile and oxygen exposure differ between scales. In lab-scale extruders, intimate contact between melt and heater surfaces accelerates oxidative degradation [55], whereas semi-industrial barrels can maintain regions of lower temperatures or reduced oxygen diffusion between cycles [56]. Under these conditions, radical chain ends generated earlier can undergo limited recombination, partially restoring polymer properties. Consequently, semi-industrial multi-cycle extrusion exhibited an initial decline of viscosity and molecular weight followed by partial recovery.

### 3.4. TGA Results

**Figure 4:**
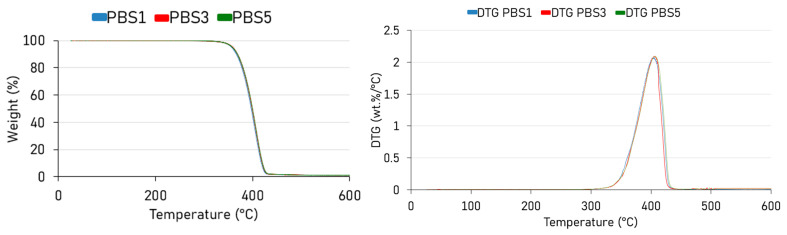
Unchanged weight loss during TGA test for all the formulations, also expressed by similar peaks in DTG.

The absence of significant changes in the thermogravimetric analysis (TGA) and its derivative of PBS after multiple reprocessing cycles (Figure 4) can be attributed to the nature of the thermal degradation processes and the stability of the polymer’s backbone under these conditions [57]. In the case of PBS, studies have shown that its thermal degradation typically occurs in a single step, beginning around 300 °C and concluding near 430 °C when under a nitrogen atmosphere [58], which is perfectly confirmed by the data of the present work. This degradation involves the breakdown of the polymer backbone, leading to significant weight loss [59].

However, the reprocessing of PBS, such as through extrusion, is conducted at temperatures significantly lower than its onset degradation temperature. For instance, processing temperatures are often around 190–230 °C. At these temperatures, while some thermo-mechanical degradation can occur, manifested as chain scission leading to the already mentioned increased melt-flow rate (MFR), the primary polymer backbone remains largely intact. This means that the fundamental thermal degradation behavior, as detected by TGA, remains unchanged across multiple reprocessing cycles.

Moreover, the degradation observed during reprocessing is often due to physical or chemical changes that do not significantly alter the thermal decomposition pathway of the polymer. Therefore, TGA curves remain consistent, indicating that the overall thermal stability of PBS is retained despite multiple processing cycles.

### 3.5. DSC Results

**Figure 5:**
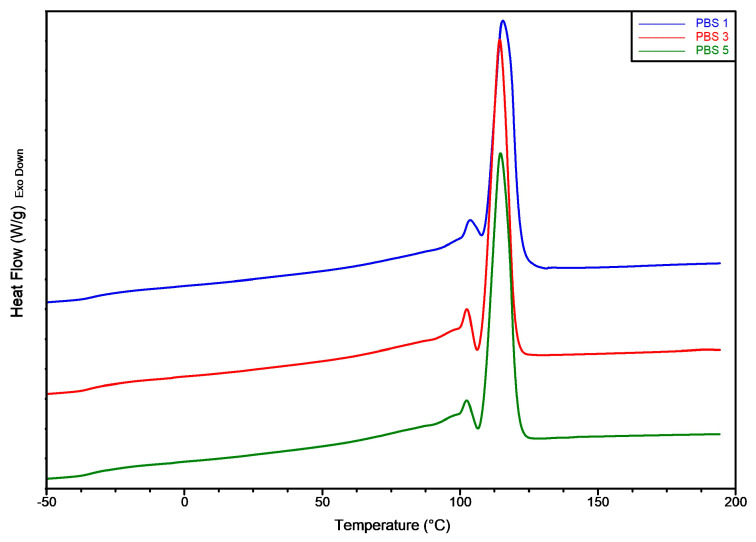
Overlay of DSC thermograms of the first heating run.

The differential scanning calorimetry (DSC) analysis of PBS subjected to one, three, and five extrusion cycles revealed only minor changes in the thermal behavior of the material, suggesting that the polymer maintained its thermal properties throughout multiple reprocessing steps (all the data are shown in Table 3 and Figure 5). The glass transition temperature (T_g_) remained practically constant, observed around −37 to −38 °C across all samples, indicating that the amorphous regions of the polymer chains retain their mobility and are not significantly influenced by thermal-mechanical degradation.

A double melting peak behaviour was observed, and it was correlated to the melt recrystallization phenomenon. In fact, according to the literature [60,61] PBS can form crystals with different stability levels; fewer stable crystals melt at lower temperatures, while more stable ones melt at higher temperatures. The melting peak of the original crystals and the recrystallized crystals can be well distinguished. In Table 4, T_m1_ refers to the main melting peak associated with the melting of the recrystallized crystals. In contrast, T_m2_ refers to the smaller melting peak related to the melting of less stable crystals. Increasing the reprocessing cycles resulted in a slight decrease in the melting temperatures [44]. This subtle shift aligns with findings in literature, which reported comparable behavior in other aliphatic polyesters like PLA, attributing it to a reduction in molecular weight following reprocessing [62]. Additionally, the melting enthalpy (ΔH_m_), which reflects the degree of crystallinity, decreased from 78.0 J/g in PBS1 to 70.1 J/g in PBS3 and remained unchanged at 70.1 J/g in PBS5. This suggests an initial decline in crystallinity due to polymer degradation and subsequent stabilization after three cycles in accordance with the results and discussion of rheological tests and the evaluation of molecular weight. This is also confirmed by the data of the crystallization percentage, which was equal in PBS3 and PBS5. The relatively constant values between PBS3 and PBS5 indicate that most of the molecular restructuring occurred during the early stages of reprocessing, beyond which the crystalline regions appeared to become less sensitive to further processing. These trends are consistent with prior studies, which demonstrate that PBS exhibits good thermal resilience and maintains its semi-crystalline nature even after repeated melt processing [63]. The degree of branching was not excessive; however, it was associated with the slight decrease in crystal formation, as the reduced mobility of the polymer chains caused by branching limited crystal formation. In fact, crystallinity decreased from 70% in PBS1 to 63% in PBS5.

### 3.6. Tensile Results

**Figure 6:**
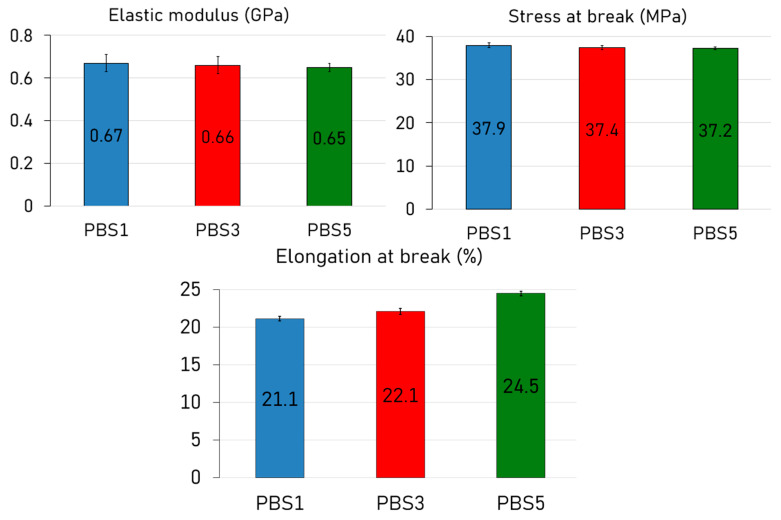
Mechanical behavior after tensile tests for PBS1, PBS3, and PBS5.

The tensile test results for PBS subjected to one, three, and five extrusion cycles revealed a remarkably stable mechanical profile (Figure 6), suggesting a limited detrimental effect of reprocessing on the structural integrity of the polymer.

The elastic modulus showed a very slight and almost negligible decrease from 0.67 GPa in PBS1 to 0.66 GPa and 0.65 GPa in PBS3 and PBS5, respectively. Similarly, the stress at break experienced only a marginal decline from 37.9 MPa in PBS1 to 37.4 MPa and 37.2 MPa in PBS3 and PBS5, respectively. These values were within experimental variability and suggest that reprocessing did not significantly compromise the properties. Interestingly, this observation is in line with the thermal analyses, where DSC and TGA confirmed minimal structural deterioration upon reprocessing. A more pronounced change was observed in the elongation at break, which slightly increased with the number of cycles: from 21.1% in PBS1 to 22.1% and 24.5% in PBS3 and PBS5, respectively. This trend might be connected with MFR data, resulting in increased chain mobility and ductility. This phenomenon has been reported in the literature for other biodegradable polyesters, such as PLA and PCL, where chain scission during processing leads to improved elongation without compromising tensile strength [64]. Overall, the mechanical performance of PBS demonstrated a high degree of resistance across multiple reprocessing cycles. The negligible changes in stiffness and strength, accompanied by a slight increase in ductility, reinforce its suitability for recycling.

### 3.7. HDT Results

**Figure 7:**
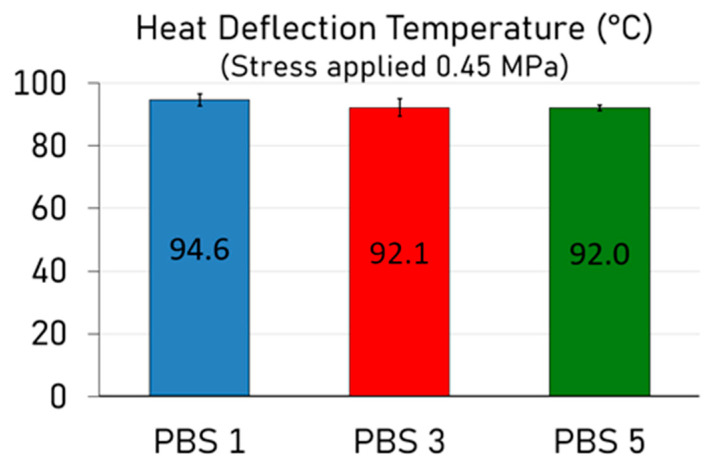
Trend of the HDT results for the reprocessed and molded PBSs.

In terms of thermomechanical performance, HDT tests were conducted under a constant applied stress of 0.45 MPa. As shown in Figure 7, the results demonstrated excellent reproducibility across all samples, with only a minimal reduction (approximately 2 °C) in the HDT values between the first and fifth processing cycles.

This slight decrease suggests a marginal loss in thermal resistance, which can be reasonably attributed to minor chain scission or slight changes in the crystallinity of the material, as previously observed in the DSC and MFR analyses. Nevertheless, all samples maintained thermal stability at temperatures exceeding 90 °C with relatively narrow error bars, indicating good consistency and reliability of the data. This stability is particularly relevant for secondary applications that require dimensionally stable materials under moderate thermal loads, such as injection-molded technical parts, which are also used for high-demand applications.

### 3.8. Impact Behavior

**Figure 8:**
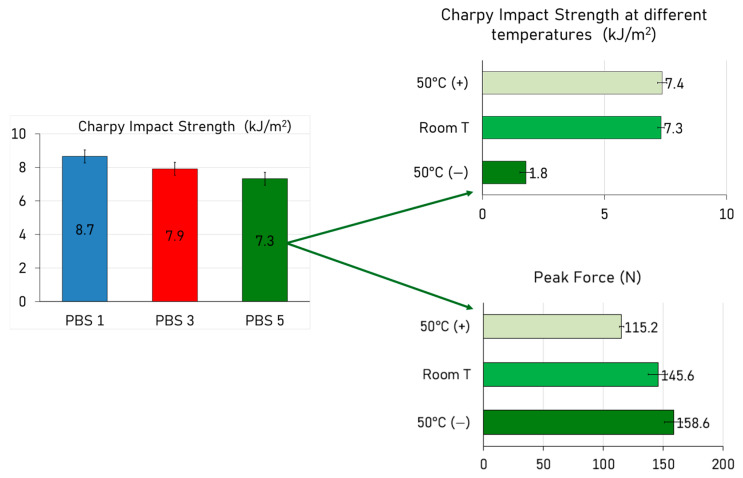
Charpy impact results as a function of temperature for all the formulations and evaluation of the effect of the temperature on the PBS recycled five times.

The Charpy impact strength of PBS (Figure 8) showed a gradual decrease with increasing reprocessing cycles, dropping from 8.7 kJ/m^2^ in PBS1 to 7.9 kJ/m^2^ in PBS3 and reaching 7.3 kJ/m^2^ in PBS5. This behavior is consistent with findings in other biodegradable aliphatic polyesters, such as PLA and PCL, where mechanical recycling similarly leads to a moderate decline in impact strength [65].

The high tensile ductility does not contradict the observed decrease in impact resistance, as these two properties are related to different types of mechanical stress. While tensile tests evaluate the material’s response to a gradual and uniaxial load, impact tests involve sudden and dynamic loading conditions. Therefore, the mechanisms governing deformation and failure in these two cases are distinct, and improvements in tensile ductility do not necessarily imply enhanced impact resistance [66].

To further assess the temperature sensitivity of the recycled material, Charpy impact tests were conducted on PBS5 at three different temperatures: 50 °C, room temperature (approximately 23 °C), and −50 °C. The results revealed a stable impact resistance between 50 °C and room temperature, with Charpy values remaining virtually unchanged. This suggests that the amorphous phase of PBS at these temperatures remains sufficiently mobile to absorb impact energy without compromising toughness. However, a dramatic drop in impact strength was observed when the test was performed at −50 °C, where the value dropped to 1.8 kJ/m^2^. At this temperature, the polymer entered a glassy state, and the amorphous regions lost their capacity to deform plastically, becoming brittle instead. As a result, the material’s ability to absorb and dissipate energy during impact was drastically reduced.

This trend was further confirmed by peak force measurements during the impact events. While the Charpy values dropped significantly at −50 °C, the peak force increased from 115.2 N at 50 °C to 145.6 N at room temperature, reaching 158.6 N at −50 °C. This behavior is typical of brittle failure mechanisms, where higher forces are recorded due to reduced deformation before fracture. The increase in peak force combined with the drop in impact energy confirmed that the material transitioned to a brittle fracture mode at low temperatures.

### 3.9. Aesthetic Properties: Color Variation

**Figure 9:**
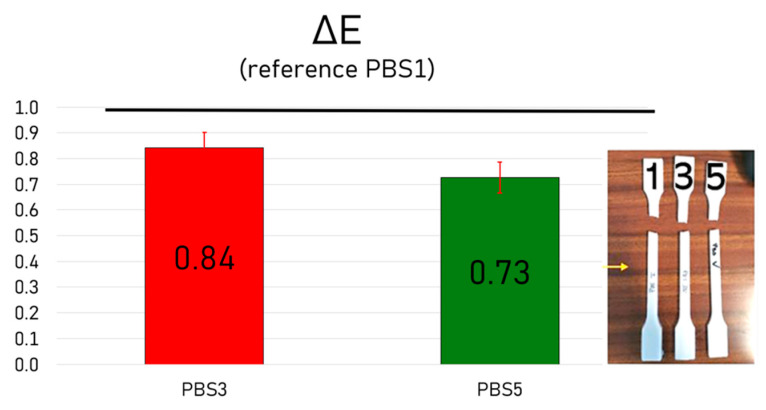
Color variation (ΔE) of specimens PBS3 and PBS5 with respect to PBS1, which was taken as reference.

To evaluate the visual appearance of PBS after multiple reprocessing cycles, colorimetric analysis was carried out using PBS1 as the reference to evaluate the color difference (ΔE) (Figure 9).

In the present study, the ΔE values recorded between PBS1 and PBS3, as well as between PBS1 and PBS5, remained consistently below 1, indicating excellent color stability throughout reprocessing.

This suggests that the thermal and oxidative stresses applied during extrusion did not significantly affect the optical properties of the material. Maintaining consistent color is especially relevant for applications where visual uniformity is critical, such as consumer goods, packaging, and biomedical devices.

## 4. Conclusions

The findings of this work confirm the strong potential of poly(butylene succinate) (PBS) to be reused in a circular production framework without significant loss of functional or visual performance. While repeated extrusion induced chain scission, as evidenced by increased MFR and reduced molecular weight and viscosity in PBS3, further reprocessing appeared to trigger secondary branching reactions that restored some of the polymer’s molecular complexity, as observed in PBS5. This self-stabilizing effect may partially mitigate the impact of thermal degradation over time.

Thermal analyses (DSC and TGA) demonstrated that the crystalline and thermal decomposition behaviors of PBS remained mainly unaffected by multiple processing cycles. Mechanical properties exhibited only marginal deterioration, with slight decreases in strength and modulus offset by enhanced elongation, indicating improved ductility. Impact strength showed moderate sensitivity to processing and a sharp transition to brittleness at low temperatures, which should be considered for cold-environment applications.

The preservation of color (ΔE < 1) and stability in thermomechanical performance (HDT > 90 °C) further affirmed that PBS retained application-relevant properties even after five extrusion cycles. These results collectively support the feasibility of mechanically recycling PBS in semi-industrial contexts, promoting its role as a durable, sustainable material in line with circular economy strategies.

## Figures and Tables

**Table 1:** Extrusion zone temperatures.

Zone	Temperature (°C)
Zone 1	110
Zone 2	135
Zone 3	150
Zone 4	155
Zone 5	155
Zone 6	150
Zone 7	145
Zone 8	140
Zone 9	135
Zone 10	135
Zone 11	130

**Table 2:** Technical notes (extrusion cycles).

Parameter	Extr. 1 (PBS1)	Extr. 2	Extr. 3 (PBS3)	Extr. 4	Extr. 5 (PBS5)
Head pressure (bar)	10	8.5	8	8	8.5
Power absorption (%)	85	82.3	81.9	81.2	82.5
Feed rate (kg/h)	15	15	15	15	15
Screw speed (rpm)	255	255	255	255	255

**Table 3:** Molecular weight and polydispersity index (PDI) after each reprocessing cycle.

Sample	M_n_ (Da)	M_w_ (Da)	PDI
PBS 1	68,300	163,640	2.395
PBS 3	74,500	151,600	2.034
PBS 5	91,300	175,800	1.925

**Table 4:** Thermal transitions and properties of PBS reprocessed one, three and five times.

Sample	T_g_ (°C)	T_m1_ (°C)	T_m2_ (°C)	ΔH_m_ (J/g)	X_c_ (%)
PBS1	−37.2	115.5	103.7	78.0	70.7
PBS3	−38.1	114.4	102.5	70.1	63.6
PBS5	−37.6	114.6	102.5	70.0	63.5

## Data Availability

The original contributions presented in this study are included in the article. Further inquiries can be directed to the corresponding author.
